# Prognostic significance of the novel nutrition-inflammation marker of lymphocyte–C-reactive protein ratio in patients with nasopharyngeal carcinoma receiving concurrent chemoradiotherapy

**DOI:** 10.3389/fnut.2023.1162280

**Published:** 2023-07-20

**Authors:** Xin Hua, Zhi-Qing Long, Si-Fen Wang, Fei Xu, Meng-Di Wang, Jia-Yi Chen, Yu-Ling Zhang, Wei-Qiong Ni, Yun-Sheng Gao

**Affiliations:** ^1^Department of Radiation Oncology, Shanghai Jiao Tong University Medical School Affiliated Ruijin Hospital, Shanghai, China; ^2^State Key Laboratory of Oncology in South China, Guangdong Key Laboratory of Nasopharyngeal Carcinoma Diagnosis and Therapy, Sun Yat-sen University Cancer Center, Guangzhou, China; ^3^Department of Endocrinology, Jiangxi Provincial People's Hospital, The First Affiliated Hospital of Nanchang Medical College, Nanchang, China

**Keywords:** LCR, nasopharyngeal carcinoma, concurrent chemoradiotherapy, prognosis, nomogram

## Abstract

**Background:**

Recent studies indicate that the novel lymphocyte–C-reactive protein ratio (LCR) is strongly associated with the survival of various tumors, but its prognostic value in nasopharyngeal carcinoma (NPC) is understudied. This study aimed to explore the relationship between LCR and overall survival (OS) in NPC and develop a predictive model.

**Methods:**

A total of 841 NPC patients who received concurrent chemoradiotherapy (CCRT) between January 2010 and December 2014 were retrospectively enrolled and randomly divided into a training cohort (*n* = 589) and a validation cohort (*n* = 252), and 122 patients between January 2015 and March 2015 were included as an additional validation cohort. Univariate and multivariate Cox analyses were performed to identify variables associated with OS and construct a predictive nomogram. The predictive accuracy of the nomogram was evaluated and independently validated.

**Results:**

The LCR score differentiated NPC patients into two groups with distinct prognoses (HR = 0.53; 95% CI: 0.32–0.89, *P* = 0.014). Multivariate analysis showed that age, T stage, N stage, EBV-DNA status, and LCR score were independently associated with OS, and a predictive nomogram was developed. The nomogram had a good performance for the prediction of OS [C-index = 0.770 (95% CI: 0.675–0.864)]. and outperformed the traditional staging system [C-index = 0.589 (95% CI: 0.385–0.792)]. The results were internally and additionally validated using independent cohorts.

**Conclusion:**

The pretreatment LCR could independently predict the overall survival in NPC patients. A novel LCR-based prognostic model of an easy-to-use nomogram was established, and it outperformed the conventional staging system in terms of predictive power. Further external verification remains necessary.

## Introduction

Nasopharyngeal carcinoma (NPC) is a relatively rare malignancy around the world but is endemic in East and Southeast Asia. More than 130,000 newly diagnosed NPC cases are reported worldwide annually, and more than 70% of these cases are locoregionally advanced (1, 2). Concurrent chemoradiotherapy (CCRT) has been widely recommended as the mainstay care for locoregionally advanced NPC (3, 4). In current clinical practice, tumor node metastasis staging is the primary and, to some extent, the only tool used for predicting prognosis and guiding treatment (5, 6). However, the survival outcome of patients at the same Tumor, Node, and Metastasis (TNM, a cancer staging system used to describe the extent or spread of solid tumors) stage differs largely (7), with up to 30% of patients in the same stage and received similar treatment regimens exhibit disease progress (8), suggesting that prognosis prediction and individualized treatment determined by anatomical staging system alone is not sufficient. Therefore, it remains imperative to explore novel markers to enhance the current traditional staging system.

Tumor-related nutrition and inflammation have been recognized as crucial factors in the development and progression of various types of cancer (9, 10). Over the past years, numerous nutritional-inflammatory prognostic indexes, including the neutrophil–lymphocyte ratio, platelet–lymphocyte ratio, the monocyte–lymphocyte ratio, the systemic immune-inflammation index, the Glasgow prognostic score, the prognostic nutritional index, and the Controlling Nutritional Status, have been established and applied clinically in various tumors including NPC (11–17). Recently, a novel nutrition-inflammation marker of the lymphocyte–C-reactive protein ratio (LCR), calculated by lymphocyte count and C-reactive protein (CRP), was found to be an effective prognostic marker in numerous tumors (18–21). However, whether the LCR score could predict survival outcomes in NPC patients remains unclear.

Herein, this study sought to explore the prognostic significance of the LCR score and establish a predictive model for individualized survival predictions in patients with NPC receiving CCRT.

## Methods

### Patients

This retrospective study enrolled consecutive NPC patients who received platinum-based CCRT between January 2010 and March 2015 at the Sun Yat-sen University Cancer Center. The following were the inclusion criteria: (i) treatment-naïve non-metastatic NPC verified by histological and radiographic evaluations; (ii) with pretreatment peripheral blood and Epstein–Barr virus (EBV) DNA examinations; (iii) with radical intensity-modulated radiotherapy plus weekly/triweekly platinum-based concurrent chemotherapy; and (iv) without any chronic inflammatory disease. All the patients updated staging according to the 8th AJCC TNM system. The participants who enrolled between January 2010 and December 2014 were randomly divided into the training and validation cohorts at a ratio of 7:3, and 122 patients who enrolled between January 2015 and March 2015 were included as an additional validation cohort. The Research Ethics Committee of Sun Yat-sen University Cancer Center approved this study, and all the patients provided written informed consent before treatment.

### Data collection and follow-up

The primary laboratory data were collected within a week of diagnosis, and clinicopathological data were obtained from patients' medical records (refer to our previously published article) (22). Real-time quantitative polymerase chain reaction was used to measure the plasma EBV-DNA levels (copies/ml) (23). Body mass index (BMI) was calculated as weight (kg)/square of the height in meters (m^2^), and patients were classified as obese (BMI ≥ 28), overweight (24 ≤ BMI < 28) and non-obese/overweight (BMI < 24). The treatment and follow-up protocols were in accordance with the guidelines previously described (23). Overall survival (OS) was defined as the time from the date of diagnosis to the date of death or last follow-up.

### Statistical analysis

Prior sample size calculations were not performed because of the lack of evidence for building prognostic models. However, the enrolled participants were 841, and the events of this study amounted to 86, with an excess rate of 10 events per variable in multivariate models, indicating sufficient evaluating power (24). The optimal cutoff value was determined by the maximally selected rank statistics with survival status as the endpoint using the “maxstat” package (25). The Kaplan–Meier method was used to produce the survival curves and compared using log-rank tests. Schoenfeld residuals were used to test the proportional hazards hypothesis. To develop and validate the prognostic model, the Cox proportional hazards model was used to perform univariate and multivariate analyses, and variables with a *p*-value of < 0.05 in the univariate analysis would be included in the multivariate analysis to identify the independent risk factors. A prognostic model was constructed using the independent risk factors identified in the multivariate analysis of the training cohort and was graphically presented as a nomogram. Harrell's concordance index (C-index) calculated by the “rms” package, time-dependent receiver operative characteristics (tROC) conducted by the “timeROC” package, and decision curve analysis (DCA) conducted by the “ggDCA” package were used to measure the models' discriminative ability in both the training and validation cohorts. C-index, calibration curve, the area under the curve (AUC) of the tROC analysis, and DCA curve were used to measure the nomogram's performance. A two-tailed *p*-value of < 0.05 was considered statistically significant. Statistical analyses were conducted using R 4.2.1.

## Results

### Patient characteristics

Table 1 shows the baseline clinicopathological characteristics of the training and validation cohorts, and they were comparable between the two groups. Supplementary Table 1 shows the baseline clinicopathological characteristics of the additional validation cohort. In total, 287 (48.7%) patients and 126 (50.0%) patients aged more than 45 years were included in the training and validation cohorts, respectively. Of the total number of patients in the training and validation cohorts, 434 (73.7%) patients were included in the former, while the latter consisted of 192 (76.2%) male patients. Most of the enrolled patients were pathologically diagnosed with histological type WHO grade III, and 200 (34.0%) patients and 78 (31.0%) patients had an EBV-DNA value of ≥4,000 copies/ml in the training and validation cohorts, respectively. Then, patients in the training cohort were divided into the high-LCR group (scored ≥1.04, *n* = 335) and the low-LCR group (scored <1.04, *n* = 254), based on the optimal LCR cutoff value of 1.04 established by the maximally selected rank statistics (Supplementary Figure 1). Using the same cutoff value of 1.04, patients in the validation cohort were also divided into the high-LCR group (scored ≥1.04, *n* = 148) and the low-LCR group (scored <1.04, *n* = 104).

**Table 1:** Patient demographics and clinical characteristics between the training and validation cohorts.

**Characteristics**	**Training cohort (*n* = 589)**	**Validation cohort (*n* = 252)**	***P***
**Age**			0.792
≥45 years	287 (48.7%)	126 (50.0%)	
< 45 years	302 (51.3%)	126 (50.0%)	
**Gender**			0.498
Male	434 (73.7%)	192 (76.2%)	
Female	155 (26.3%)	60 (23.8%)	
**Histological type**			0.225
WHO grade I/II	7 (1.19%)	6 (2.38%)	
WHO grade III	582 (98.8%)	246 (97.6%)	
**HGB**			0.782
< 113 g/L	18 (3.06%)	7 (2.78%)	
113–151 g/L	382 (64.9%)	158 (62.7%)	
≥151 g/L	189 (32.1%)	87 (34.5%)	
**LDH**			0.183
≥245 U/L	31 (5.26%)	20 (7.94%)	
< 245 U/L	558 (94.7%)	232 (92.1%)	
**ALB**			0.577
≥40 g/L	536 (91.0%)	233 (92.5%)	
< 40 g/L	53 (9.0%)	19 (7.5%)	
**T stage**			0.288
T1	33 (5.60%)	8 (3.17%)	
T2	105 (17.8%)	55 (21.8%)	
T3	365 (62.0%)	151 (59.9%)	
T4	86 (14.6%)	38 (15.1%)	
**N stage**			0.631
N0	52 (8.83%)	28 (11.1%)	
N1	319 (54.2%)	132 (52.4%)	
N2	190 (32.3%)	77 (30.6%)	
N3	28 (4.75%)	15 (5.95%)	
**BMI**			0.904
≤ 24 kg/m^2^	353 (59.9%)	152 (60.3%)	
24–28 kg/m^2^	201(34.1%)	87(34.5%)	
≥28 kg/m^2^	35 (5.9%)	13 (5.2%)	
**EBV-DNA**			0.442
< 4,000 copies/ml	389 (66.0%)	174 (69.0%)	
≥4,000 copies/ml	200 (34.0%)	78 (31.0%)	
**LCR**			0.673
< 1.04	254 (43.1%)	104 (41.3%)	
≥1.04	335 (56.9%)	148 (58.7%)	

WHO, World Health Organization; HGB, hemoglobin; LDH, serum lactate dehydrogenase levels; ALB, albumin; BMI, body mass index; EBV-DNA, Epstein-Barr virus DNA; LCR, lymphocyte–C-reactive protein ratio.

### Prognostic value of LCR score for OS in NPC

**Figure 1:**
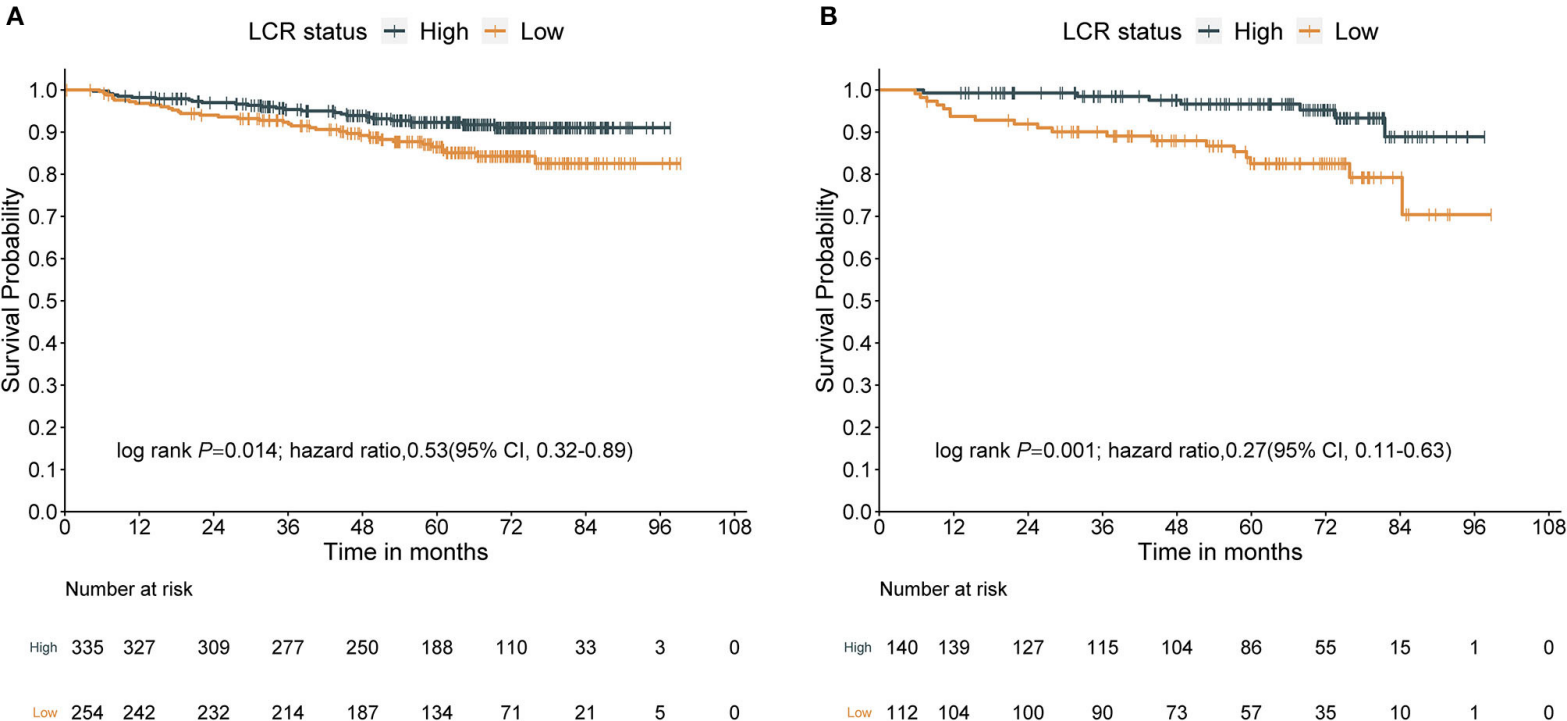
Survival curves obtained with Kaplan–Meier analysis between different LCRs. Groups (the HRs reported were unadjusted). **(A)** Survival curves in the training cohort. **(B)** Survival curves in the validation cohort. LCR, lymphocyte–C-reactive protein ratio; HR, hazard ratios; CI, confidence interval.

In the whole cohort of 841 patients, the median follow-up was 64.1 months (IQR: 58.3–76.5 months), and the median OS was 62.5 months (IQR: 46.6–74.8 months). There were 86 death events observed in the whole cohort, with 60 in the training cohort and 26 in the validation cohort. In the whole cohort, the 1-, 3-, and 5-year OS rates were 97.4%, 94.4%, and 91.1%, respectively. In the training cohort, the 1-, 3-, and 5-year OS rates were 97.6%, 94.2%, and 90.8%, respectively. In the validation cohort, the 1-, 3- and 5-year OS rates were 96.8%, 94.8%, and 91.7%, respectively. There was no significant difference in OS between the training and validation cohorts (Supplementary Figure 2, *P* = 0.96). Kaplan–Meier curves demonstrated that patients in the high-LCR group had significantly better survival than those in the low-LCR group in the training, validation, and additional cohorts (Figure 1A, HR = 0.53; 95% CI: 0.32–0.89, *P* = 0.014; Figure 1B, HR = 0.27; 95% CI: 0.11–0.63, *P* = 0.001; Supplementary Figure 3, HR = 0.28; 95% CI: 0.09–0.91, *P* = 0.024).

### Univariate and multivariate Cox regression analyses of OS in NPC

Univariate and multivariate Cox regression analyses were conducted both in the training and validation cohorts. In the training cohort, variables that met the prespecified significance threshold (*P* < 0.05) in the univariate Cox model, including age, T stage, N stage, EBV-DNA status, and LCR score, were entered into the multivariate Cox regression model. A multicollinearity diagnostic test was conducted by calculating the variance inflation factors (VIFs) of the above variables (all VIFs < 10), indicating that no severe multicollinearity exists. According to the proportional hazards diagnostic plots (Supplementary Figure 4), the multivariable modeling satisfied the proportional hazards assumption. The results of multivariate modeling demonstrated that age, N stage, EBV-DNA status, and the LCR score were independently associated with OS for patients with NPC receiving concurrent chemoradiotherapy in the training cohort (Table 2) and validation cohort (Table 3).

**Table 2:** Univariate and multivariate Cox regression analyses of overall survival in the training cohort.

**Characteristics**	**Univariate analysis**	***P***	**Multivariate analysis**	***P***
	**Hazard ratio (95%CI)**		**Hazard ratio (95%CI)**	
**Age**				
≥45 years	1		1	
< 45 years	0.642 (0.414–1.000)	0.049	0.638 (0.408–0.995)	0.048
**Gender**				
Male	1			
Female	0.768 (0.450–1.312)	0.334		
**Histological type**				
WHO grade I/II	1			
WHO grade III	2.402 (0.757–7.617)	0.137		
**HGB**				
< 113 g/L	1			
113–151 g/L	2.182 (0.301–15.824)	0.440		
≥151 g/L	3.084 (0.422–22.527)	0.267		
**LDH**				
≥245 U/L	1			
< 245 U/L	0.723 (0.315–1.661)	0.445		
**ALB**				
≥40 g/L	1			
< 40 g/L	1.457 (0.622–3.412)	0.386		
**T stage**				
T1	1		1	
T2	2.943 (0.380–22.800)	0.301	2.748 (0.354–21.340)	0.334
T3	4.020 (0.555–29.120)	0.168	3.749 (0.516–27.267)	0.192
T4	7.516 (1.009–56.010)	0.049	6.395 (0.853–47.940)	0.071
**N stage**				
N0	1		1	
N1	1.531 (0.542–4.323)	0.421	1.656 (0.583–4.705)	0.344
N2	2.907 (1.033–8.181)	0.043	2.906 (1.014–8.334)	0.047
N3	4.630(1.425–15.039)	0.011	3.963 (1.185–13.255)	0.025
**BMI**				
≤ 24 kg/m^2^	1			
24–28 kg/m^2^	0.619 (0.328–1.166)	0.138		
≥28 kg/m^2^	0.791 (0.243–2.568)	0.696		
**EBV-DNA**				
< 4,000 copies/ml	1		1	
≥4,000 copies/ml	1.734 (1.117–2.694)	0.014	1.651 (1.041–2.618)	0.033
**LCR**				
< 1.04	1		1	
≥1.04	0.530 (0.317–0.885)	0.015	0.612 (0.386–0.971)	0.037

Hazard ratios estimated by Cox proportional hazards regression. All statistical tests were two-sided. WHO, World Health Organization; HGB, hemoglobin; LDH, serum lactate dehydrogenase levels; ALB, albumin; BMI, body mass index; EBV-DNA, Epstein-Barr virus DNA; LCR, lymphocyte–C-reactive protein ratio.

**Table 3:** Univariate and multivariate Cox regression analyses of overall survival in the validation cohort.

**Characteristics**	**Univariate analysis**	***P***	**Multivariate analysis**	***P***
	**Hazard ratio (95%CI)**		**Hazard ratio (95%CI)**	
**Age**				
≥45 years	1		1	
< 45 years	0.574 (0.360–0.916)	0.020	0.569 (0.354–0.915)	0.020
**Gender**				
Male	1			
Female	0.783 (0.450–1.363)	0.388		
**Histological type**				
WHO grade I/II	1			
WHO grade III	2.813 (0.886–8.937)	0.08		
**HGB**				
< 113 g/L	1			
113–151 g/L	2.013 (0.277–14.620)	0.489		
≥151 g/L	2.768 (0.377–20.300)	0.317		
**LDH**				
≥245 U/L	1			
< 245 U/L	0.898 (0.328–2.461)	0.835		
**ALB**				
≥40 g/L	1			
< 40 g/L	1.133 (0.492–2.612)	0.769		
**T stage**				
T1	1		1	
T2	2.455 (0.311–19.380)	0.394	2.300 (0.291–18.210)	0.430
T3	3.566 (0.491–25.890)	0.209	3.225 (0.443–23.493)	0.248
T4	7.324 (0.983–54.580)	0.050	5.570 (0.741–41.881)	0.095
**N stage**				
N0	1		1	
N1	1.530 (0.542–4.319)	0.422	1.704 (0.601–4.835)	0.316
N2	2.889 (1.027–8.130)	0.045	2.957 (1.033–8.465)	0.043
N3	72.006 (12.510–414.455)	< 0.001	35.680 (5.937–214.423)	< 0.001
**BMI**				
≤ 24 kg/m^2^	1			
24–28 kg/m^2^	0.638 (0.377–1.081)	0.095		
≥28 kg/m^2^	0.778 (0.281–2.152)	0.629		
**EBV-DNA**				
< 4,000 copies/ml	1		1	
≥4,000 copies/ml	2.481 (1.572–3.917)	0.001	1.754 (1.081–2.844)	0.023
**LCR**				
< 1.04	1		1	
≥1.04	0.417 (0.260–0.666)	0.001	0.561 (0.344–0.916)	0.021

Hazard ratios estimated by Cox proportional hazards regression. All statistical tests were two-sided. WHO, World Health Organization; HGB, hemoglobin; LDH, serum lactate dehydrogenase levels; ALB, albumin; BMI, body mass index; EBV-DNA, Epstein-Barr virus DNA; LCR, lymphocyte–C-reactive protein ratio.

### Development of a novel prognostic model based on LCR

**Figure 2:**
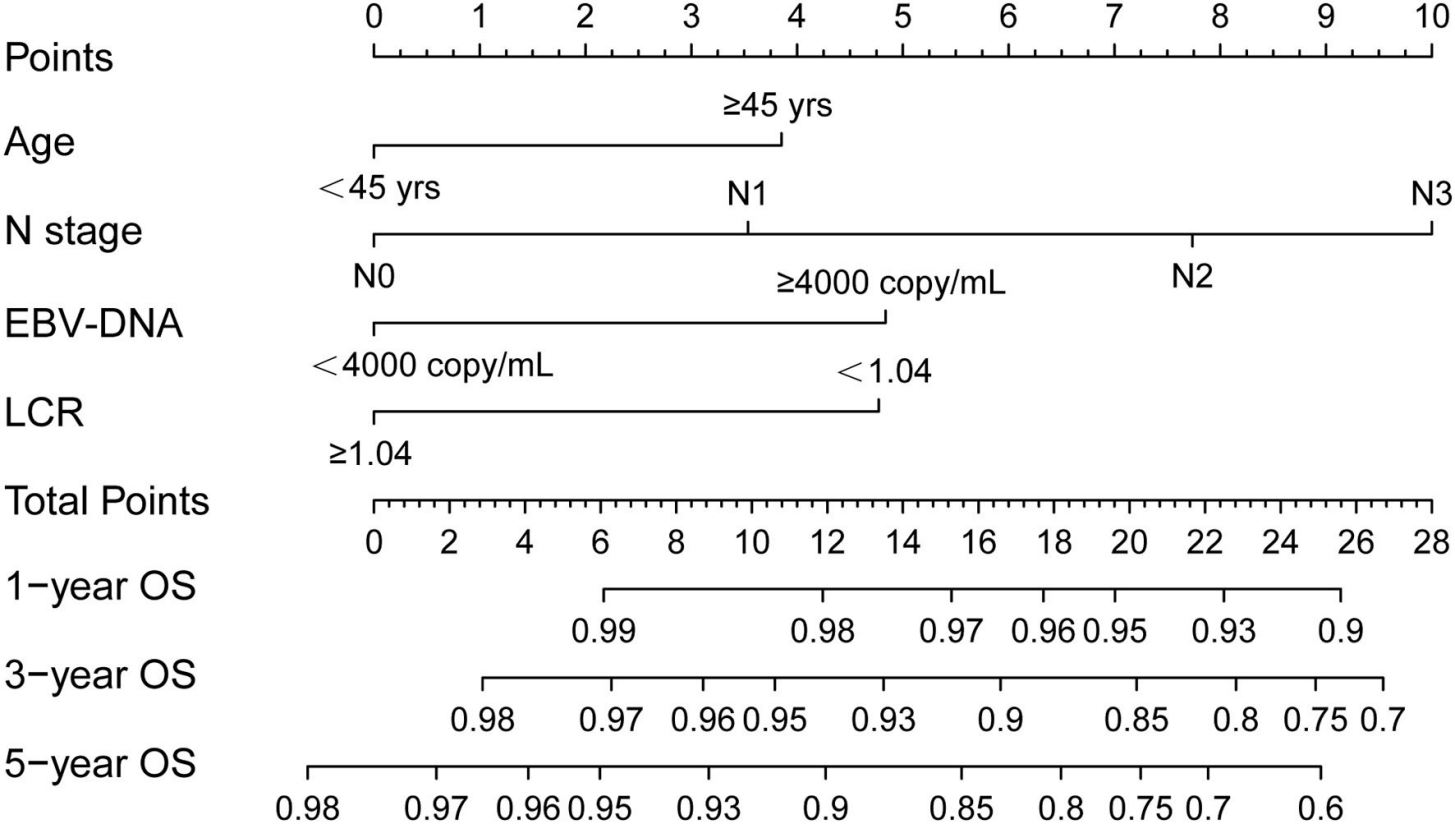
Nomogram of the current prognostic model for individualized survival predictions. OS, overall survival; LCR, lymphocyte–C-reactive protein ratio; EBV-DNA, Epstein-Barr virus DNA.

A novel prognostic model of a nomogram for individual survival prediction at 1-, 3-, and 5-year was developed, based on the above four independent indicators from the multivariate modeling (Figure 2). Before the implementation of CCRT, each patient's total score could be calculated by adding the scores from each of the four prognostic factor subclasses, and the 1-, 3-, and 5-year OS probabilities could be forecasted by positioning the total score on the survival rate scale. For example, one patient who was 40 years old was presented with N3 stage, EBV-DNA of ≥4,000 copies/ml, and an LCR score of <1.04; hence, the total point was 0 + 10 + 4.8 + 4.75 = 19.55, and the corresponding 1-, 3-, and 5-year OS probabilities were 95%, 86%, and 77%, respectively.

### Assessment of predictive performance of the prognostic model

**Figure 3:**
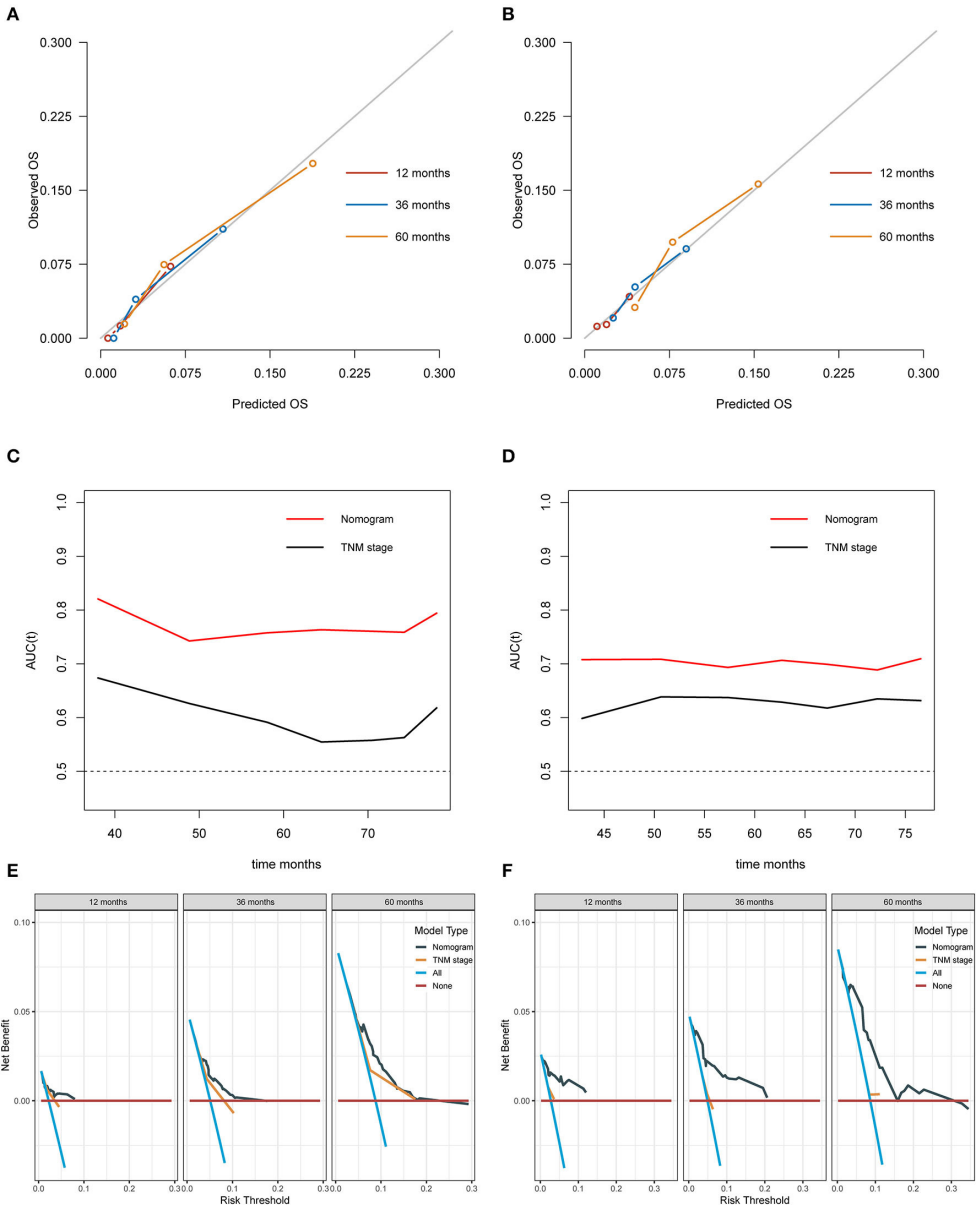
Assessment of predictive performance of the prognostic model. **(A)** Calibration plot of the nomogram model at 1, 3, and 5 years in the training cohort. **(B)** Calibration plot of the nomogram model at 1, 3, and 5 years in the validation cohort. **(C)** Time-independent ROC curves compared the predictive accuracy of the current model and the traditional TNM stage in the training cohort. **(D)** Time-independent ROC curves compared the predictive accuracy of the current model and the traditional TNM stage in the validation cohort. **(E)** DCA curves compared the net benefit rate of the current model and the traditional TNM stage in the training cohort. **(F)** DCA curves compared the net benefit rate of the current model and the traditional TNM stage in the validation cohort. OS, overall survival; AUC, area under the curve; TNM, tumor node metastasis.

The generated prognostic model showed well-discriminative ability with a good C-index of 0.770 (95% CI: 0.675–0.864), 0.698 (95% CI: 0.623–0.774), and 0.727 (95% CI: 0.492–0.961) in the training, validation, and additional validation cohorts, compared with the C-index of 0.589 (95% CI: 0.385–0.792), 0.641 (95% CI: 0.532–0.749), and 0.644 (95% CI: 0.410–0.879) of traditional staging system; 0.662 (95% CI: 0.534–0.791), 0.646 (95% CI: 0.440–0.853), and 0.564 (95% CI: 0.282–0.845) of age; 0.694 (95% CI: 0.575–0.812), 0.651 (95% CI: 0.448–0.854), and 0.540 (95% CI: 0.254–0.825) of EBV-DNA; and 0.555 (95% CI: 0.351–0.758), 0.602 (95% CI: 0.493–0.710), and 0.651 (95% CI: 0.417–0.886) of the N stage, respectively. The calibration plots (the Y-axis represents the actual observed survival, while the X-axis represents the nomogram predicted survival) for the 1-, 3-, and 5-year OS present good agreement between predicted OS and observed OS in the training (Figure 3A), validation (Figure 3B), and additional validation (Supplementary Figure 5A) cohorts. The prognostic accuracy of this prognostic model for customized OS was evaluated using time-dependent ROC curves, and it outperformed the conventional tumor node metastasis (TNM) stage in the training (Figure 3C), validation (Figure 3D), and additional validation (Supplementary Figure 5B) cohorts. Also, the DCA curves demonstrated that the application of nomogram provided a better prediction effect than the TNM stage in the training (Figure 3E), validation (Figure 3F), and additional validation (Supplementary Figure 5C) cohorts.

## Discussion

In this study, we investigated that the pretreatment LCR could independently predict the OS in NPC patients, and a novel LCR-based prognostic model of an easy-to-use nomogram was established. The findings were also reproducible in the validation cohort. Given that the traditional anatomical TNM staging system alone is not sufficient for prognosis prediction in NPC patients who underwent CCRT, the novel nutrition-inflammation marker of LCR and LCR-based prognostic model could serve as a simplified, affordable, easy-to-obtain, non-invasive, and readily promotive biomarker to clinically make individualized prognostic recommendations in this heterogeneous patient population.

NPC is biologically highly heterogeneous, with quite different outcomes for the same staging patients receiving similar regimens of CCRT (7). In recent years, highly sophisticated gene examination and liquid biopsy have been used to interpret the comprehensive molecular mechanisms of NPC to discriminate its heterogeneity and prognosis (26–29). However, these tools are currently expensive and have complicated testing procedures with unreliable repeatability; therefore, they are not yet widely applied in clinical practice. Since the term “biomarker” was initially proposed by The National Institutes of Health Biomarkers Definition Working Group (30), increasing studies have explored and developed numerous novel prognostic markers in NPC. Nowadays, the optimal prognostic biomarkers are generally recognized as signatures that could identify an individual's prognosis independent of traditional classifications (i.e., the TNM staging system) to improve clinical survival outcomes and are affordable, easily accessible, simplified, and use standard approaches, which can be scaled up on a large scale. Although there is numerous established evidence of the TNM staging system for evaluating the risk of disease progress and prognosis in NPC patients, the huge heterogeneity of prognostic outcomes in patient groups with the same stage and received similar treatment regimens highlights the urgent need for novel efficient biomarkers to identify patients at high risk for long-term clinical outcomes in NPC.

In this study, the novel nutrition-inflammation marker of LCR was demonstrated as being significantly related to survival outcomes in NPC patients who underwent CCRT. Those individuals with high systemic inflammation and malnutrition risk factors were more likely to have a poor prognosis and an insufficient response to radiation and chemotherapy than patients without these risk factors. Our findings were consistent with those of earlier studies investigating certain nutritional or inflammatory markers in NPC (31–33). Poorer survival outcomes in NPC have been linked to higher CRP levels and severe lymphopenia risks. Our findings suggested that evaluations of the nutritional and inflammatory status of the LCR score may accurately reflect the genuine tumor condition and forecast the effectiveness of CCRT in NPC. An under-appreciated sub-population of patients who have a low LCR score may need individualized treatment.

However, the molecular mechanisms underpinning the association between LCR status and prognosis are still not fully understood. Complex connections between the tumor and host immunological and inflammatory responses are associated with cancer-related nutrition and inflammation, and these interactions could serve as potential targets for cancer therapies (9, 34). The mechanisms of the prognostic significance of the LCR status in NPC may be partially explained by the physiological and pathologic roles of lymphocytes and CRP in tumors. By causing cytotoxic cell death and obstructing tumor cell proliferation and migration, lymphocytes play a significant role in tumor immune surveillance and defense (34). Malnutrition could decrease the prognosis of patients by weakening the host's immune system functioning and cell-mediated immunity, which is crucial for the host's ability to prevent cancer and is indicated by the lymphocyte level (35). Malnutrition has been linked to worse prognosis for a variety of malignant cancers, due to the increased adverse effects, treatment pauses, reduced chemotherapy intensity, decreased radiation sensitivity and/or chemotherapy sensitivity, and compromised immune function of the host (36–38). Cancer-related inflammation is a recognized hallmark of cancer that regulates essentially all stages of malignancy, including susceptibility, initiation, progression, dissemination, and death (39). Circulating immune cells, circulating cytokines, tiny inflammatory proteins, and acute-phase proteins are all mediators of systemic inflammation (34). CRP is an acute-phase protein that is largely produced in hepatocytes in reaction to proinflammatory cytokines before being released into the bloodstream. Moreover, there is a vicious facilitation between inflammation and malnutrition (40). Therefore, it is crucial to identify patients at high risk of infection and malnutrition, thus providing anti-infective and nutritional interventions as soon as possible are essential to improve clinical outcomes.

Apart from the LCR score, the age, N stage, and EBV-DNA status were independently associated with OS for patients with NPC receiving CCRT. Age is one of the important prognostic factors in many tumors including NPC, and previous studies have confirmed that elder patients have progressively worse survival in NPC (41, 42). Our previous study also found that patients over 45 years old had a high risk to develop low skeletal muscle mass (malnutrition) (43). Theoretically, an increased risk of malnutrition and radiotherapy/chemotherapy-related toxicities may have occurred with advancing age due to the accumulation of cellular damage, declining immune function, and impaired organ function (42). The N stage was also found to be associated with the nutritional status of NPC patients. For example, Du et al. found that a higher N stage was related to severe weight loss in NPC patients, and Wei et al. revealed that nodal metastasis status was correlated to the patient's nutritional index (44, 45). Chronic inflammation induced by EB virus infection has been shown to play a crucial role in the progression and invasiveness of NPC (46, 47). Also, Tang et al. found that EBV-DNA was related to the patient's nutritional index in NPC, and many studies demonstrated adding the prognostic value of EBV-DNA to nutritional and inflammatory biomarkers (48–50).

## Limitations

There are certain unavoidable limitations to this cohort study. First, this study was conducted retrospectively, and there exists an inherent potential selection bias. Second, the patient cohorts used in this study are not necessarily typical of all cancer patients who are diagnosed and treated at the institution because the study population was centered on patients who had adequate measurements for the chosen markers. Moreover, there was no external validation conducted for the prognostic model due to our inability to obtain high-quality data from other centers. Therefore, further multicenter external validation would be necessary to strengthen our findings. Additionally, the LCR status can be influenced by several clinical situations, and it can change over time. Therefore, we will collect more data to undertake dynamic analysis to produce more results, and we are also planning further prospective studies to confirm these findings.

## Conclusion

The pretreatment LCR could independently predict the overall survival in NPC patients. A novel LCR-based prognostic model of an easy-to-use nomogram was established, and it outperformed the conventional staging system in terms of predictive power. The LCR score may enable oncologists to estimate individualized survival outcomes more accurately, thus supporting the appropriate pre-CCRT and post-CCRT management of NPC patients. Further external verification of LCR and LCR-based prognostic model remains necessary.

## Data availability statement

The original contributions presented in the study are included in the article/Supplementary material, further inquiries can be directed to the corresponding authors.

## Ethics statement

The Research Ethics Committee of Sun Yat-sen University Cancer Center approved this study, and all the patients provided written informed consent before treatment.

## Author contributions

XH performed the literature search and designed the study. XH, Z-QL, S-FW, FX, M-DW, J-YC, Y-LZ, W-QN, and Y-SG participated in the analysis and interpretation of data. XH and Y-SG developed an early draft. All authors contributed to the manuscript revision and approved the final version before submission.
