# Supplementary material for: Prognostic significance of the novel nutrition-inflammation marker of lymphocyte–C-reactive protein ratio in patients with nasopharyngeal carcinoma receiving concurrent chemoradiotherapy
Source: Front Nutr. 2023 Jul 20;10:1162280. doi: 10.3389/fnut.2023.1162280 (PMC10399231; doi:10.3389/fnut.2023.1162280)

**Supplementary Materials**

**Table S1**. Patient demographics and clinical characteristics between the training and validation cohorts.

| Characteristic | Additional validation cohort (n=122) |
| --- | --- |
| Age |  |
| ≥45 years | 64 (52.5%) |
| ＜45 years | 58 (47.5%) |
| Gender |  |
| Male | 95 (77.9%) |
| Female | 27 (22.1%) |
| Histological type |  |
| WHO Ⅰ/Ⅱ | 2 (1.6%) |
| WHO Ⅲ | 120 (98.4%) |
| HGB |  |
| ＜113 g/L | 4 (3.3%) |
| 113-151 g/L | 80 (65.6%) |
| ≥151 g/L | 38 (31.1%) |
| LDH |  |
| ≥245 U/L | 9 (7.4%) |
| ＜245 U/L | 113 (92.6%) |
| ALB |  |
| ≥40 g/L | 107 (87.7%) |
| ＜40 g/L | 15 (12.3%) |
| T stage |  |
| T1 | 5 (4.1%) |
| T2 | 34 (27.9%) |
| T3 | 71 (58.2%) |
| T4 | 12 (9.8%) |
| N stage |  |
| N0 | 15 (12.3%) |
| N1 | 61 (50.0%) |
| N2 | 34 (27.9%) |
| N3 | 12 (9.8%) |
| BMI |  |
| ≤24 kg/m^2^ | 65 (53.3%) |
| 24-28 kg/m^2^ | 53(43.4%) |
| ≥28 kg/m^2^ | 4 (3.3%) |
| EBV-DNA |  |
| ＜4000 copy/ml | 78 (63.9%) |
| ≥4000 copy/ml | 44 (36.1%) |
| LCR |  |
| ＜1.04 | 48 (39.3%) |
| ≥1.04 | 74 (60.7%) |

Abbreviations: WHO = World Health Organization; HGB = hemoglobin; LDH = serum lactate dehydrogenase levels; BMI = body mass index; EBV-DNA = Epstein-Barr virus DNA; LCR = lymphocyte to C-reactive protein ratio.

**Figure S1.** Definition of the cutoff value of 1.04 for the LCR score according to maximally selected log-rank statistics.


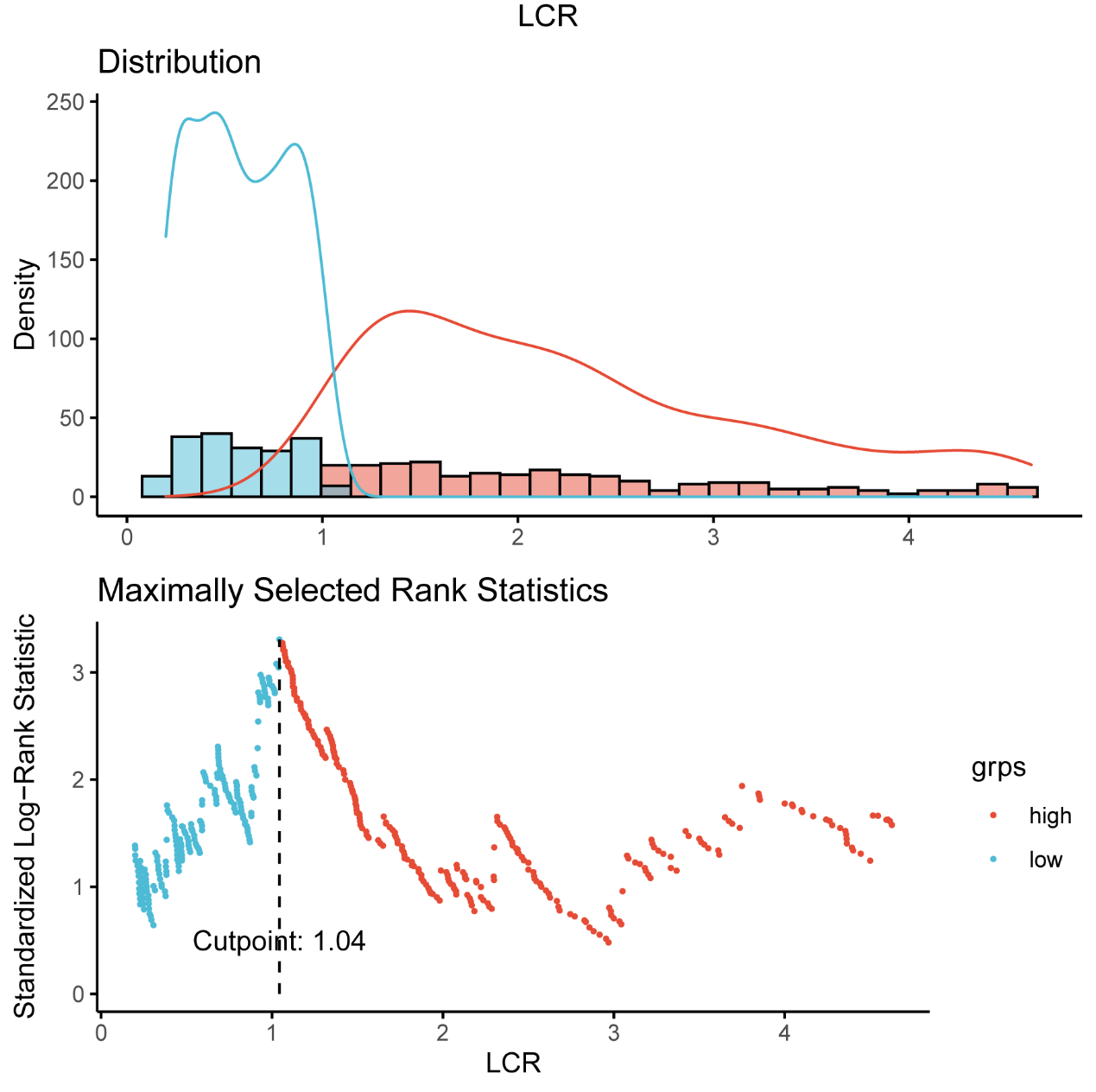


**Figure S2.** Survival curves obtained with Kaplan-Meier analysis between different cohorts.


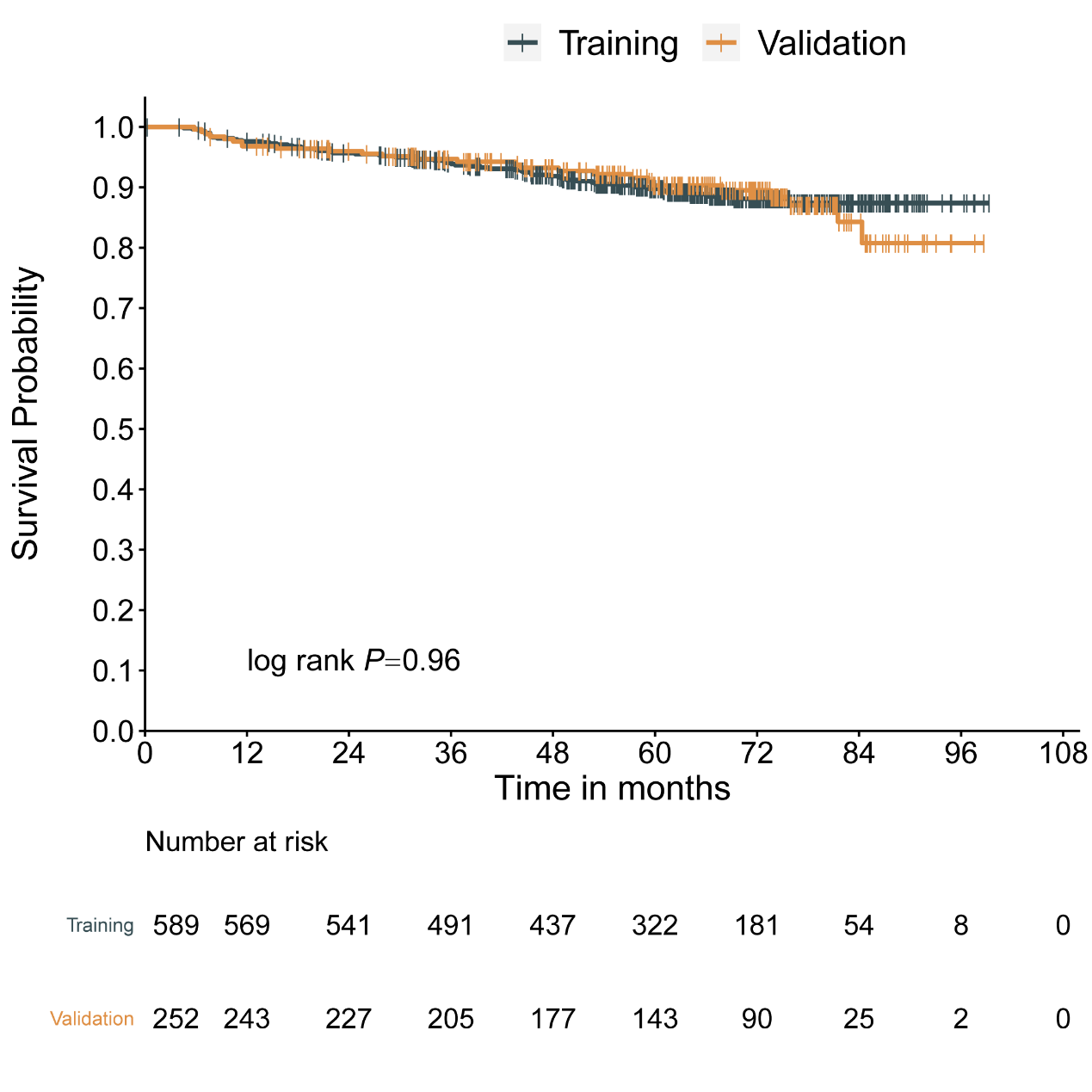


**Figure S3**: Survival curves obtained with Kaplan-Meier analysis between different LCR Groups (the HRs reported were unadjusted) in the additional validation cohort.

Abbreviations: LCR = lymphocyte-C-reactive protein ratio; HR = hazard ratios; CI = confidence interval.


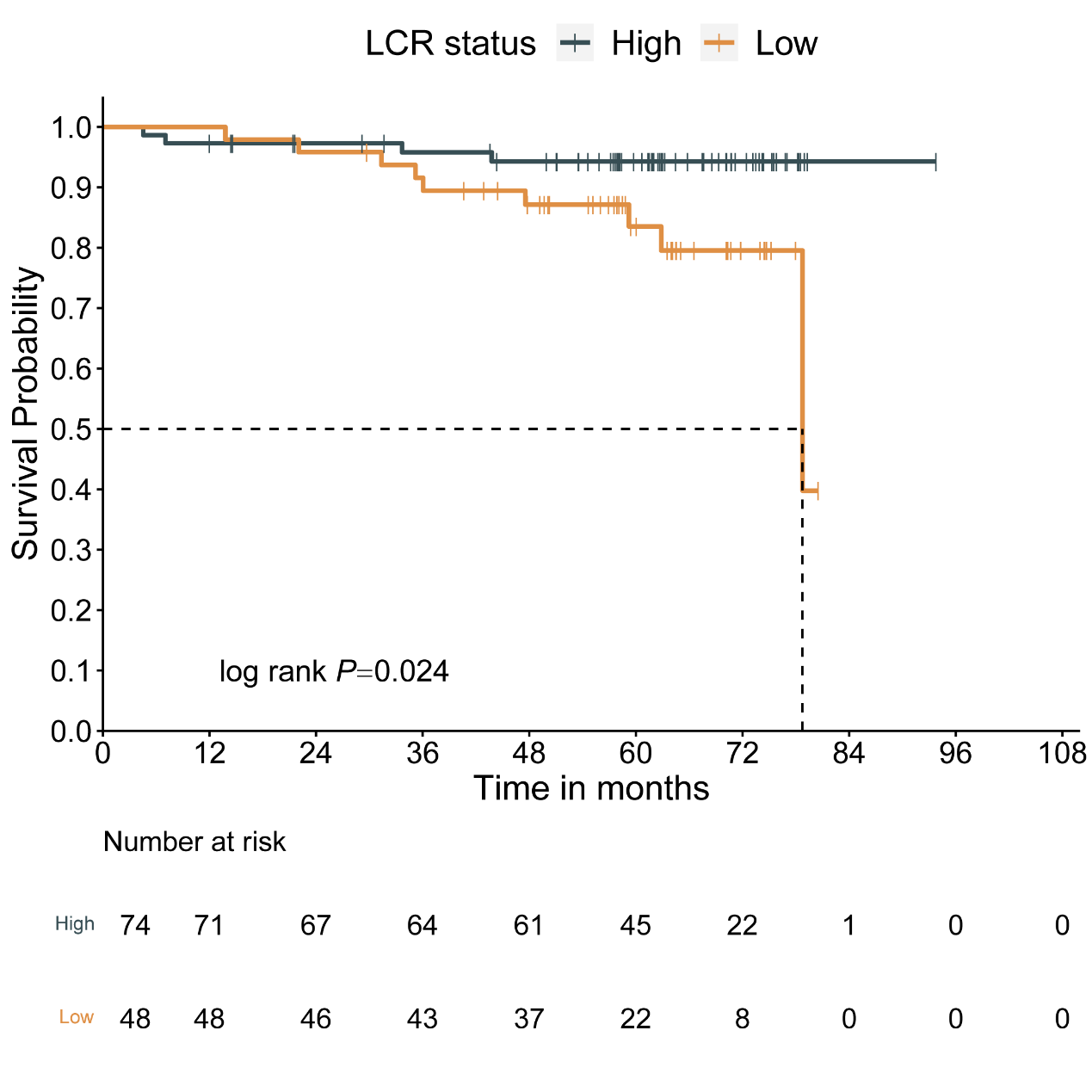


**Figure S4.** Proportional hazards diagnostic plots of multivariable Cox modeling.


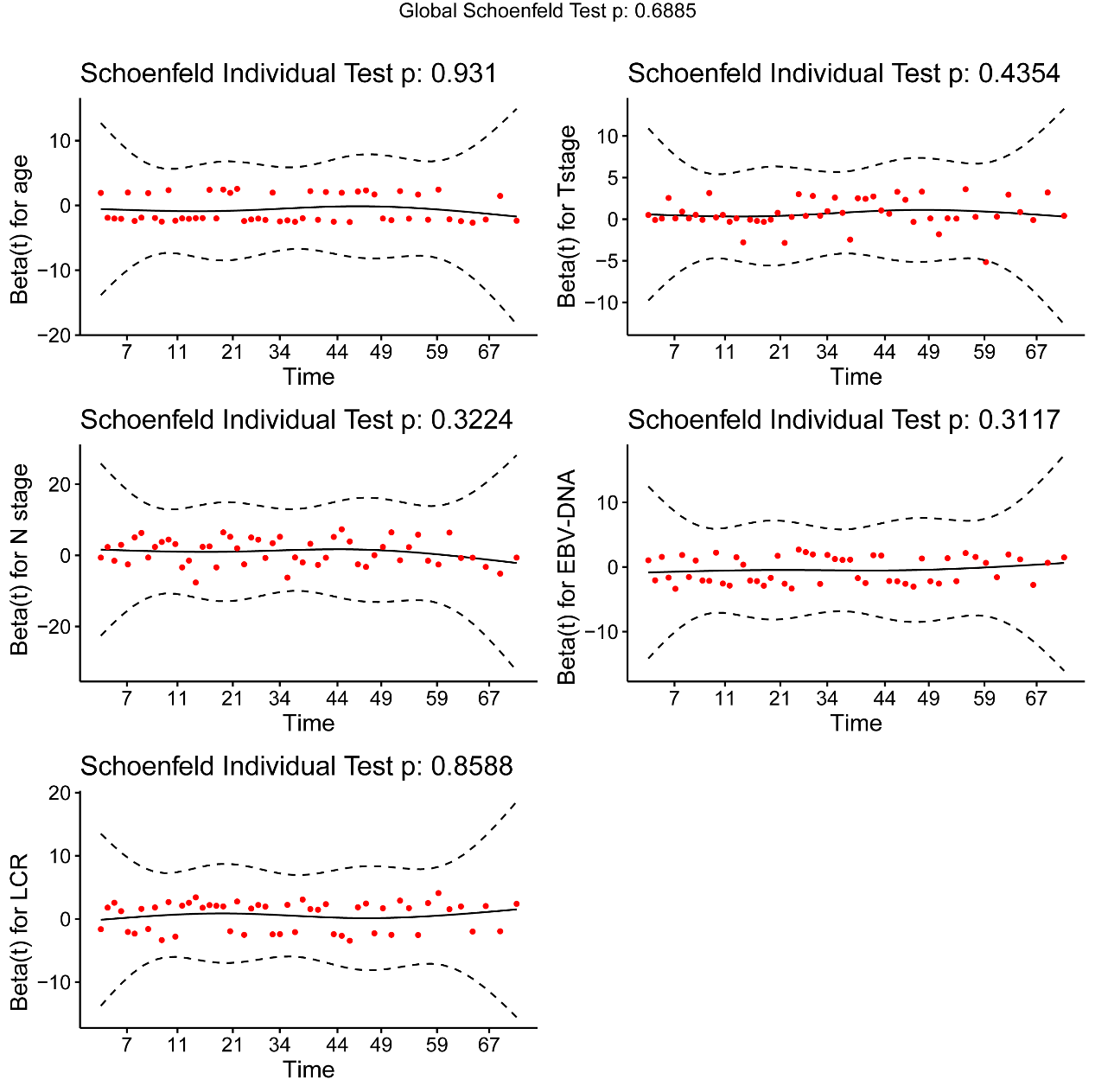


**Figure S5.** Assessment of predictive performance of the prognostic model in the additional validation cohort.

(A) Calibration plot of the nomogram model at 1- , 3-, and 5-year.

(B) Time-independent ROC curves compared the predictive accuracy of the current model and the traditional TNM stage.

(C) DCA curves compared the net benefit rate of the current model and the traditional TNM stage.

Abbreviations: OS = overall survival; AUC = area under curve; TNM = tumor-node-metastasis.


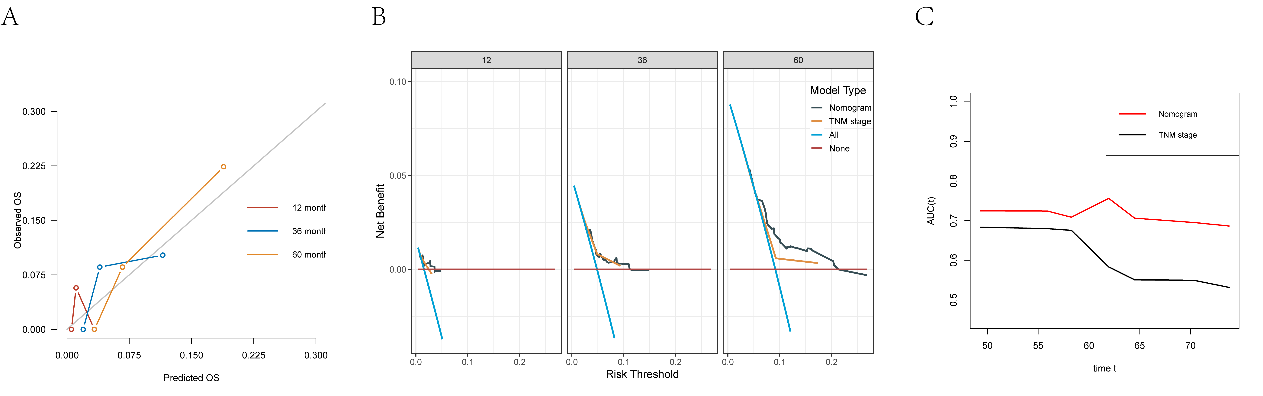

Supplement: Supplementary file 1 [file Data_Sheet_1.docx]
